# Precision Soil Moisture Monitoring Through Drone-Based Hyperspectral Imaging and PCA-Driven Machine Learning

**DOI:** 10.3390/s25030782

**Published:** 2025-01-28

**Authors:** Milad Vahidi, Sanaz Shafian, William Hunter Frame

**Affiliations:** School of Plant and Environmental Sciences, Virginia Polytechnic Institute and State University, Blacksburg, VA 24061, USA; miladvahidi@vt.edu (M.V.); whframe@vt.edu (W.H.F.)

**Keywords:** root zone soil water content, drone-based imaging, PCA-driven data analysis, monitoring models

## Abstract

Accurately estimating soil moisture at multiple depths is essential for sustainable farming practices, as it supports efficient irrigation management, optimizes crop yields, and conserves water resources. This study integrates a drone-mounted hyperspectral sensor with machine learning techniques to enhance soil moisture estimation at 10 cm and 30 cm depths in a cornfield. The primary aim was to understand the relationship between root zone water content and canopy reflectance, pinpoint the depths where this relationship is most significant, identify the most informative wavelengths, and train a machine learning model using those wavelengths to estimate soil moisture. Our results demonstrate that PCA effectively detected critical variables for soil moisture estimation, with the ANN model outperforming other machine learning algorithms, including Random Forest (RF), Support Vector Regression (SVR), and Gradient Boosting (XGBoost). Model comparisons between irrigated and non-irrigated treatments showed that soil moisture in non-irrigated plots could be estimated with greater accuracy across various dates. This finding indicates that plants experiencing high water stress exhibit more significant spectral variability in their canopy, enhancing the correlation with soil moisture in the root zone. Moreover, over the growing season, when corn exhibits high chlorophyll content and increased resilience to environmental stressors, the correlation between canopy spectrum and root zone soil moisture weakens. Error analysis revealed the lowest relative estimation errors in non-irrigated plots at a 30 cm depth, aligning with periods of elevated water stress at shallower levels, which drove deeper root growth and strengthened the canopy reflectance relationship. This correlation corresponded to lower RMSE values, highlighting improved model accuracy.

## 1. Introduction

Understanding soil moisture dynamics is essential for enhancing agricultural productivity, optimizing water resource management, and promoting environmental sustainability [1]. Accurate soil moisture estimation is crucial, as it directly influences plant growth, nutrient uptake, and root development [2,3]. Also, as agriculture faces increasing pressures from climate change and population growth, effective moisture management becomes vital for ensuring optimal crop health and maximizing yields [4].

Traditional soil moisture measurement relies on in situ sampling and gravimetric analysis, which, while precise, are labor-intensive and limited in spatial coverage. These conventional methods can provide accurate point measurements but fail to capture the broader spatial variability of soil moisture across agricultural landscapes. This limitation emphasizes the need for more scalable and efficient approaches to monitor soil moisture over larger areas [5,6].

Advancements in remote sensing technologies have revolutionized soil moisture estimation by providing non-invasive, large-scale alternatives to traditional measurement methods. Satellite-based remote sensing, through systems like the Soil Moisture Active Passive (SMAP) and the European Space Agency’s Sentinel missions, can monitor soil moisture dynamics over vast regions. These satellite-based systems utilize microwave and optical sensors to capture surface moisture data, facilitating timely interventions in irrigation and resource management [7].

The capabilities of satellite remote sensing for extensive coverage are well-recognized, yet the spatial resolution provided by such platforms can sometimes be insufficient for detailed local assessments [8].

This limitation is especially pertinent in precision agriculture, where understanding variability in soil moisture at a granular level is crucial for optimizing irrigation strategies and enhancing crop management. Here, drone-based remote sensing technologies prove invaluable, offering high-resolution imagery that facilitates more precise analyses of soil moisture across smaller field segments.

Concerning the efficacy of different sensor types, it is important to clarify that while traditional multispectral and thermal sensors are widely used for various remote sensing applications, they do have limitations in soil moisture estimation. Multispectral sensors, which collect data across several specific wavelength bands, are adept at identifying surface characteristics. However, they often lack the sensitivity required to detect subtle spectral differences that indicate moisture content at various soil depths. On the other hand, thermal sensors are effective at measuring surface temperatures, but their ability to estimate soil moisture is often compromised by overlying vegetation, which can obscure or distort the thermal signature of the underlying soil. These sensors are predominantly tailored for surface observations and may not provide reliable data on moisture content deeper within the soil profile.

Therefore, while satellite images remain a fundamental resource for broad-scale environmental monitoring, their lower resolution and the limitations of certain sensors necessitate supplementary data sources for detailed moisture analysis. UAV technology, equipped with advanced imaging capabilities, addresses these gaps by delivering the high-resolution, site-specific data required for effective precision agriculture practices. This integrated approach harnesses the strengths of both satellite and drone technologies, providing a comprehensive toolset for soil moisture management and agricultural planning.

Hyperspectral sensors, however, offer a significant advantage over multispectral and thermal sensors. By capturing continuous spectral data across a wide range of wavelengths, hyperspectral sensors provide the fine spectral resolution necessary for detecting subtle features that correlate with soil moisture content. This capability enables more precise identification of specific wavelengths related to moisture levels, enhancing the accuracy of soil moisture estimates, particularly beneath vegetative canopies.

Incorporating Principal Component Analysis (PCA) into this framework (hyperspectral images) will further enhance the effectiveness of hyperspectral data analysis. PCA serves as a powerful dimensionality reduction technique, allowing us to isolate the most informative wavelengths that significantly contribute to soil moisture estimation. By reducing the complexity of the spectral data while retaining essential information, PCA improves the efficiency of subsequent analyses and modeling efforts, ensuring a more precise understanding of soil moisture dynamics.

Integrating drone-based hyperspectral sensors with PCA and machine learning techniques presents an innovative approach to developing robust, data-driven models for estimating root-zone soil moisture. While machine learning has been utilized in soil moisture estimation studies, the combination of PCA-applied hyperspectral data captured by drones remains an underexplored area. PCA enhances hyperspectral data analysis by reducing dimensionality and isolating the most significant wavelengths that correlate with soil moisture. This facilitates more efficient processing by machine learning algorithms, leading to more accurate predictive models.

A notable gap in the current research is the integration of time-series hyperspectral imagery with extensive ground-truth data collected throughout the agricultural season. This combination facilitates the analysis of temporal variations in soil moisture and their interactions with plant responses, offering a more comprehensive and dynamic perspective on moisture dynamics. In addition, most existing studies on soil moisture estimation focus primarily on surface soil moisture, usually within the top 5 cm of the soil profile [9,10,11]. However, understanding moisture levels at deeper layers (e.g., depths of 10 and 30 cm) is crucial for root growth and effective irrigation management. Accurately estimating soil moisture at these depths can support improved agricultural practices and boost crop resilience, particularly during periods of drought or water stress.

Our study aims to bridge this gap through a novel experimental design that captures moisture variability under both irrigated and non-irrigated conditions. This approach highlights how moisture fluctuations affect plant health over time by leveraging the canopy’s spectral signature as an indicator. The main objectives of this study are to determine whether a correlation exists between root zone water content and canopy reflectance and to identify the depth at which this relationship is most significant. Additionally, we aim to pinpoint the specific wavelengths that are most effective for soil moisture estimation, thereby enhancing our understanding and capabilities in this vital area of agricultural science.

## 2. Materials

### 2.1. Study Area

**Figure 1:**
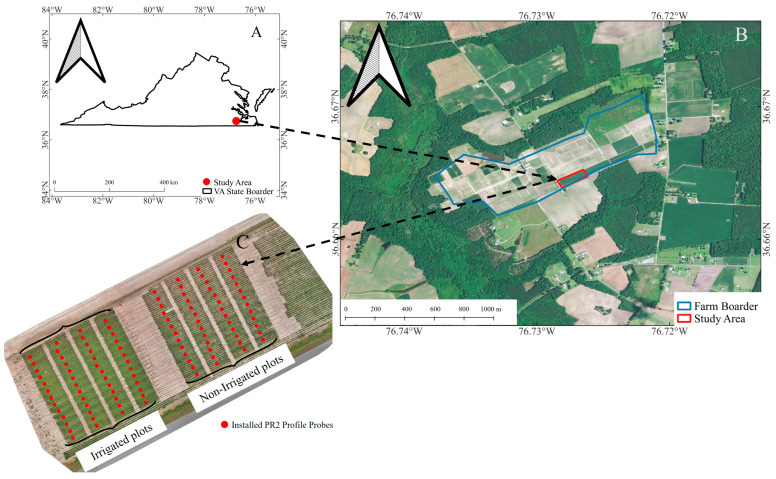
Overview of the location of study area. (**A**) Study area location on Virginia state border, (**B**) showing farm and case study borders; (**C**) orthomosaic generated from Mavic 3 Pro RGB images with installed Pro2 tubes.

This study was carried out in the spring and summer of 2023 at the Virginia Tech Tidewater Agricultural Research Extension Center (TAREC) located in Virginia, USA. The research site is a 50 by 100 m rectangular field situated within the larger expanse of TAREC, at geographic coordinates 36.6649206° N and −76.7271206° W. Positioned 19 m above sea level, this prime location features a sandy soil composition and is nearly level, with a gentle slope not exceeding 1%, and it is primarily used for growing corn. Detailed visual documentation of the study site, including an orthomosaic image produced using Pix4DMapper software 4.8.0 and RGB photographic data captured with a Mavic 3 Pro drone, is presented in Figure 1.

### 2.2. Experimental Design

Figure 1 illustrates the experimental design implemented in the study area, which was divided into irrigated and non-irrigated sections, each consisting of 52 plots. Geometrically, each plot consists of four rows, each measuring 0.76 m in width and 9.144 m in length. The study site was lightly strip-tilled in the spring near planting for seedbed preparation. A standard hybrid selected from the Virginia (VA) corn state trials (Progeny 9714^®^ cultivar, 114-day maturity) was seeded at a 32K seeding rate. Pre-plant fertilizers were applied, followed by side-dress rates determined by liquid sources such as urea ammonium nitrate (UAN 46%), Monoammonium Phosphate (MAP), Ammonium Sulfate (AMS) (24%), and potash for pre- and side-dress fertility management. The plots were allocated to two primary treatments: irrigated and non-irrigated. The irrigated plots received regular irrigation through a linear system within specified periods, with each receiving one inch of water on 9 June, 23 June, 20 July, and 3 August to ensure consistent soil moisture levels throughout the growing period. Within each treatment, thirteen different micronutrient applications were applied at the V6 growing stage, when the sixth leaf collar becomes visible, marking a significant phase of vegetative growth, with each application consisting of 56 kg ha^−1^ of phosphorus (P), potassium (K), and 11 kg ha^−1^ of sulfur (S) supplied through granular AMS (24%), Muriate of Potash (MOP) (60%), MAP (52%), and urea (46%). Each micronutrient application had four replications, resulting in a total of 104 plots. Also, both treatments have similar soil type (sandy soil) and soil surface texture as well as soil chemical content regarding the soil laboratory test conducted before planning. This test includes 15 soil cores from each plot to a depth of 30 cm, analyzing them for key soil nutrients such as P, K, calcium (Ca), magnesium (Mg), S, pH, and electrical conductivity (EC) following the Mehlich-1 weak acid procedure.

### 2.3. Hyperspectral Drone Flights and Ground Sampling

UAV-based hyperspectral remote sensing offers imagery with unparalleled spatial and spectral detail, serving as a comprehensive and systematic approach for the enhanced observation of the Earth’s surface. Over the past decade, the application of UAV hyperspectral remote sensing in terrestrial and marine projects has seen significant growth. Within this framework, efforts are underway to monitor the soil moisture by utilizing spectrum information of the plant’s canopy. This project utilizes the RESONON (a company located in United States that designs and builds hyperspectral imaging systems and software) Pika-L hyperspectral camera with a push broom imagery system over 400–1100 nm, adaptable in field applications, where it is mounted on a DJI Matrice 600 Hexacopter Aerial Drone. DJI is a Chinese technology company that manufactures and distributes drones and related imaging devices. The system comprises three main components: a ground station with software (provided by DJI company) for mission planning and parameter setting (including FOV, shutter speed, framerate, etc.; Table 1), a remote controller, and a DJI Ronin MX gimbal stabilizer holding the sensor on the drone.

To ensure superior image quality during flight missions, the DJI Ronin MX gimbal stabilizer was employed to mitigate rotational movements of the sensor about the X, Y, and Z axes. The stabilizer was set to “car-mount” mode, a feature of the Ronin MX, which maintains the sensor in a fixed nadir position, ensuring it is oriented downward towards the ground regardless of any drone deviations. This configuration is particularly effective in counteracting disturbances such as wind or shifts in the drone’s path during U-turns, thus maintaining the consistency of the sensor’s positioning throughout the data collection process.

The missions were conducted on 7 specific dates throughout the growing season to maintain uniform data collection intervals. The flights were conducted on 15 June and 30 June (V6–V8), 11 July (V8), 20 July (V10), 30 July (VT/R1), 2 August (R1), and 14 August (R2).

The decision to conduct a time series flight over corn fields on different dates is driven by several factors. Firstly, the varying biomass density serves as an indicator of plant water uptake and transpiration, influencing soil moisture levels. Secondly, the flights enable the mapping of root zones and the identification of subsurface features related to changing root density, root depth, and distribution during different phases of corn growth. In essence, canopy spectrum variation and flying over different canopy conditions contribute to more comprehensive and improved model training, resulting in a more generalizable model. To provide further explanation on corn growing stages, corn progresses through several pivotal growth stages, starting around the V4 stage where the plant stands 8 to 12 inches tall, with the growing point still protected beneath the soil surface. By the V6–V8 stages, occurring 4 to 6 weeks after germination, the growing point rises above the soil, making it vulnerable to environmental threats like hail, frost, or wind. This phase marks significant developments, including the dominance of the nodal root system and the start of a rapid growth phase at V7, with the plant reaching about 24 inches by V8. The subsequent V9–V11 stages initiate around 6 to 8 weeks after emergence, characterized by steady growth, rapid dry matter accumulation, and the internal development of the tassel, although it remains hidden. During this period, new leaves emerge every 2 to 3 days, and ear shoots form. Entering the critical VT stage around 9 to 10 weeks after emergence, the plant must successfully pollinate; this is when the tassels become fully visible and silks emerge, with pollen shedding over the next week or two. Following this, the R1 silking stage sees the start of pollination from the base to the tip of the ear. By the R2 blister stage, about 12 days post-silking, the kernels form blisters filled with clear fluid and darken silks, entering vulnerable phases where stress can significantly impact yield. Transitioning into the R3 milk and R4 dough stages, the kernels turn yellow and then develop a dough-like consistency, with moisture levels gradually reducing from 80% to about 70%

As mentioned, the time series of hyperspectral sensor flights were conducted on various dates spanning from June to August. The UAV missions were consistently planned with specific parameters to maintain uniformity in data collection. On all the dates, flights were carried out at a steady speed of approximately 7 miles per hour at an altitude of 50 m. The lateral distance between each pass of the UAV was kept in a way to create at least 20% side overlap between the data cubes. This limited overlap was chosen to facilitate the identification of corresponding points across image pairs, essential for effective image registration and subsequent mosaicking. Typically, hyperspectral imaging demands substantial memory for storage and processing; therefore, excessive overlap, which is primarily necessary for creating digital surface models, was deemed unnecessary. Regarding image resolution, both the pixel size in the X-dimension (Px) and the pixel size in the Y-dimension (Py) were set to 5 cm for all flights. This consistent approach in mission planning ensured that the resulting dataset would be temporally coherent, facilitating an accurate analysis of changes over the specified time frame.

In the field data collection process, PR2 Profile tubes were installed over each plot (at the second row of each plot shown in Figure 1) within the experimental treatments. The PR2 Profile sensor, renowned for its precision in soil moisture measurement, was employed for ground moisture sample acquisition. The PR2 sensor’s capability to capture moisture content at various depths (10 cm and 30 cm) allowed for a comprehensive understanding of the vertical distribution of soil moisture within each experimental plot. The soil moisture in all the tubes was measured at the same time as flying the Mavic3T drone. To minimize the probable error in data collection, we measured the soil moisture by replicating three times and rotating the sensor in three parts of 120 degrees, and the final moisture at each plot was an average of three replications over three rotations.

## 3. Methodology

### 3.1. Preprocessing of Hyperspectral Images

**Figure 2:**
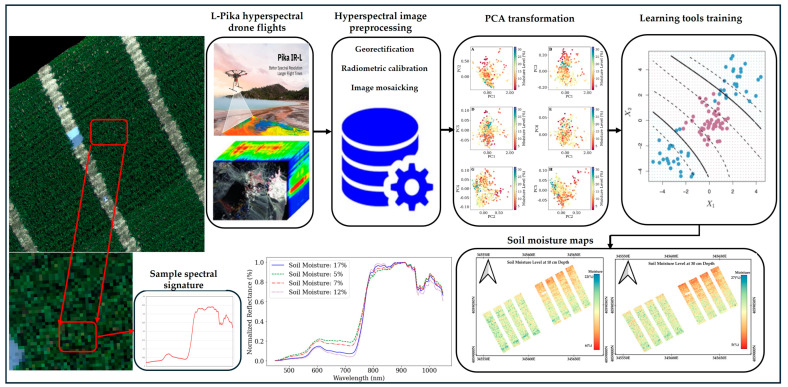
The flowchart of the methodology, PCA (principal component analysis), SVR (support vector regression), GBM (gradient boosting model), and ANN (artificial neural network).

The preprocessing of hyperspectral images is the first step of this study, as shown in Figure 2. The preprocessing of hyperspectral images is a meticulous process, essential for ensuring the accuracy and usability of the data. This procedure can be segmented into five distinct steps: conversion of raw digital numbers (DN) to radiance, transformation of radiance to reflectance values, georectification, mosaicking, and georeferencing. Initially, raw DN values captured by the sensor were converted to radiance in real-time by the flight computer, utilizing a manufacturer-supplied calibration file. This ensures that all data cubes outputted from the sensor were in radiance values. Following this, the radiance data cubes were processed using the Georectify plugin on Spectronon Pro software, specifically designed by Resonon for georectification purposes. The software allows for the incorporation of a digital terrain model to enhance georectification accuracy. To optimize computational efficiency and manage data volume, image pixels were downscaled to a 10 cm resolution.

Subsequently, the georectified radiance values underwent conversion to reflectance values. This was accomplished by referencing targets with known reflectivity placed within the study field, featuring two panels of differing gray levels that reflect 12% and 24% across all spectral bands. To further refine the data, spectral reflectance curves were smoothed using a Savitzky–Golay filter. The Savitzky–Golay filter is a technique known for its efficacy in smoothing noisy data without significantly distorting the signal [12]. For our study, we employed a filter with a kernel size of 5 and a polynomial order of 3, following recommendations provided by the sensor manufacturer. Geometric corrections were then applied to the radiometrically corrected data. Individual flight lines were mosaicked, with a 20% side overlap between consecutive lines facilitating image-to-image registration. This step is critical to align corresponding points across overlapping regions, effectively eliminating horizontal and vertical shifts between flight lines and ensuring a seamless mosaic of the data cubes.

The final phase of preprocessing involved georeferencing the comprehensive mosaic. Six ground control points (GCPs), strategically positioned across the field, were used for this purpose. The precise latitude and longitude coordinates for these GCPs were determined using a dual-frequency Emlid-Reach GPS unit with fixed status, providing accuracy within a few centimeters. Emlid is a company in Budapest, Hungary, that makes GNSS receivers, including the Reach RS3, Reach M2, and Reach RS2. These comprehensive preprocessing steps were systematically applied across all the hyperspectral image datasets in the time series, establishing a robust foundation for subsequent analyses and interpretations. The subsequent phase in the preprocessing step involves extracting the spectral values from the plant canopy. This was conducted by first opening all the data cubes in the QGIS 3.20.2 software. Within this software, a series of small polygons are delineated over each plot. The creation of these carefully sized polygons is critical; their dimensions are chosen to ensure that only the spectral data or the pixels from the canopy are captured while effectively excluding any shadowed pixels that may be present due to adjacent taller plants. Once these polygons were in place, the pixel values contained within them, which correspond to the spectral information of each plot’s canopy, were extracted and organized into a structured datasheet. This standard dataset was then ready for the further processing stages of detailed analysis.

### 3.2. Principal Component Analysis (PCA)

In this study, we utilized PCA to process hyperspectral imaging data, focusing on dimensionality reduction and feature extraction. This method is pivotal for compressing the extensive datasets obtained from hyperspectral imaging, ensuring that the critical information is preserved while simplifying the dataset for analysis. PCA transforms the original correlated spectral bands into a reduced number of uncorrelated variables, known as principal components (PCs). These components are organized such that the initial few encapsulate most of the variability found in the original dataset, thereby enhancing the efficiency of data analysis and interpretation processes.

The procedure for applying PCA to the hyperspectral images commenced with the standardization of the spectral bands. This critical first step ensures each band has a zero mean and a unit standard deviation, preventing any bias towards bands with naturally higher variances [13].

Standardization of a reflectance value $x_{i}$ in band *i* is performed as follows:

(1) $$z_{i}=\frac{x_{i}-\mu_{i}}{\sigma_{i}}\;$$

where $\mu_{i}$ and $\sigma_{i}$ denote the mean and standard deviation of band *i*, respectively.

The next step involves computing the covariance matrix to understand the variance and covariance across the spectral bands:

(2) $$cov\left(i,j\right)=\frac{1}{n-1}\overset{n}{\underset{k=1}{\sum }}\sum \left(Z_{ik}-\bar{Z_{i}}\right)\left(Z_{jk}-\bar{Z_{j}}\right)$$

Here, *n* represents the number of pixels, $Z_{ik}$ and $Z_{jk}$ are the standardized reflectance values for bands *i* and *j* for pixel *k*, and $\bar{Z_{i}}$ and $\bar{Z_{j}}$ are the means of the standardized values for bands *i* and *j*.

Eigenvalue decomposition: This crucial phase involves decomposing the covariance matrix to extract the eigenvalues and eigenvectors, which inform the directions and magnitudes of variance captured by the PCs [14].

The critical task of selecting the number of PCs to retain was guided by the scree plot analysis. The scree plot visually represents the eigenvalues in descending order, facilitating the identification of the ‘elbow point’. This point indicates when the incremental explained variance begins to decrease significantly, thereby determining the optimal number of PCs to capture the essential information in the dataset efficiently [15].

The hyperspectral data are then transformed into the principal component space using the selected eigenvectors [16]:

(3) Y=ZE

In this equation, *E* represents the matrix of eigenvectors, *Z* is the matrix of standardized data, and *Y* is the transformed data in the PC space. This results in a new dataset with uncorrelated dimensions, effectively compressing the hyperspectral data into a more manageable form while retaining the most significant variance.

In this study, PCA was employed as a technique to extract the most significant features from the hyperspectral imaging data for soil moisture estimation. The reason behind this approach lies in PCA’s adeptness at identifying and extracting PCs that capture the highest variance within the dataset. By prioritizing these PCs (principal components), we effectively extract the most informative variables that are closely associated with variations in soil moisture levels or can cause clusters of different levels of soil moisture in a feature space. This focus on the major variance-yielding components ensures that the dimensionality of our data is reduced without sacrificing critical information that can be leveraged for accurate soil moisture prediction.

### 3.3. Learning Tools

#### 3.3.1. Support Vector Machine (SVM)

Support Vector Regression (SVR) extends the concepts of Support Vector Machines (SVMs) from classification [17,18] to regression tasks [19]. It aims to predict continuous values by finding a function that deviates minimally from the actual target values for all training data while maintaining a balance between model complexity and prediction accuracy.

SVR seeks to fit the best line or hyperplane that predicts continuous target values, employing an ε-insensitive loss function to measure prediction errors. This function only penalizes errors exceeding a defined margin, ε, encouraging a model that is as flat as possible. In Linear SVR, the relationship is modeled directly, while non-linear relationships utilize kernel functions to map input data into higher-dimensional spaces where linear regression can be applied. Mathematically, SVR aims to minimize the following equation:

(4) $$\frac{1}{2}{\left\|w\right\|}^{2}+C\sum \left(\xi_{i}+\xi_{i}^{*}\right)$$

(5) $$y_{i}-w^{T}x_{i}-b\leq \epsilon +\xi_{i}\;w^{T}x_{i}+b-y_{i}\leq \epsilon +\xi_{i}^{*}\;\xi_{i},\xi_{i}^{*}\geq 0,\;\;for\;all\;i$$

subject to certain conditions where w is the weight vector, *b* is the bias, *C* is the regularization parameter, and ξi, $\xi_{i}^{*}$ are slack variables for managing deviations beyond the ε threshold. For non-linear data, SVR uses kernel functions such as polynomial (with different desired degree), Radial Basis Function (RBF), or sigmoid to perform operations in a transformed feature space without explicitly mapping the data to this space, enabling efficient handling of complex relationships. In our study, we utilized the Support Vector Machine (SVM) regression implementation provided by the scikit-learn library in Python. This choice was made to leverage the robust and efficient computational algorithms available in scikit-learn, a widely respected toolkit for machine learning tasks. To tailor the SVM regression model to our specific dataset and research objectives, we manually adjusted the model’s internal parameters [20].

#### 3.3.2. Random Forest (RF)

Random Forest (RF) regression, introduced by Tin Kam Ho, is a non-parametric, supervised, ensemble machine learning algorithm that builds predictions using a collection of decision trees [18,21,22]. By leveraging multiple decision trees rather than a single model, RF harnesses ensemble learning techniques to achieve reliable and accurate predictions, making it a popular choice for both regression and classification tasks. The core objective of RF is to generate a “forest” by combining several decision trees, typically through bootstrap aggregation, or “bagging”. A key advantage of RF is its resilience against overfitting, even with numerous features, eliminating the need for feature pre-selection when training the model [23].

Furthermore, RF has two distinct advantages over other statistical models: relative robustness against noise and the ability to recognize optimal and informative features. It means that RF can achieve its best performance even if too many or ineffective features are included in the input vector. The algorithm’s performance is entirely dependent on the parameters that must be predefined by experts for the design or training of the forest. The parameters tuned in RF include the number of trees, splitting criteria, and controlling growth limitation, known as the depth of decision trees, inside. A random forest regression will be modeled as follows: initialization of N and criteria for splitting and depth, using bootstrap aggregated (or bagged) decision trees to build multiple decision trees by repeatedly resampling training data with replacement (the trees could be constructed by different subsets of features), calculating the output of each tree, and obtaining the average of estimation for the final value. In this study, the number of estimators, maximum number of features, and maximum depth are of the parameters that have been tuned using the grid search strategy in the Keras library in the scikit-learn library in Python 3.7 [20].

#### 3.3.3. Gradian Boosting (XGBoost)

Gradian Boosting (XGBoost) is another type of decision tree-based ensemble algorithm and an improved version of the boosting technique that utilizes the gradient method to strengthen the performance of a weak model. Due to its ability to optimize the computational speed, efficiency, and performance of a model, this algorithm has become popular in learning subjects and has specifically been used for either regression or classification problems. Compared to a simple decision tree-based method (random forest algorithm that applies the bagging technique to construct a full decision tree in a sequential process), in the XGBoost algorithm, full decision trees are built in an iterative and parallel process in which a strong model is collectively generated by gradient descent and specific objective function [24]. The process of model construction follows a level-wise strategy, scanning across gradient values and using a partial sum of errors to evaluate the quality of splits at every possible split in the training set. Despite other boosting algorithms, instead of training the best model possible on the data similar to the case with traditional methods, thousands of models on subsets of the training dataset are trained, then the best-performing model is selected using a voting strategy. Self-tree purring, resistance to avoid overfitting, built-in cross-validation, and parallelization are of the advantages of the XGBoost method over other boosting algorithms. Despite its advantages over random forest, obtaining the most optimal model of XGBoost can be difficult, as the algorithm includes many inner parameters that need to be tuned for a specific problem. In this study, the XGBoost in scikit-learn library was trained via grid search strategy in Python, in which ‘colsample_bytree’, ‘learning_rate’, ‘n_estimators’, ‘subsample’, and ‘max_depth’ were optimized in a defined numerical range [20].

#### 3.3.4. Artificial Neural Network (ANN)

Artificial Neural Networks (ANNs) are non-parametric and supervised machine learning methods that attempt to imitate the pattern of information processing in a human brain to model complicated problems for prediction or deciding. Some advantages of neural networks over other regression models include the ability to model non-linear or unknown relationships between variables, robustness in dealing with noisy inputs, the ability to generalize input variables, and the lack of the need for variable-specific assumptions. On the other hand, complexity in training an optimal model, being sensitive to overfitting effects, and designing a sufficient architecture are some of the disadvantages of neural network models that affect the algorithm’s performance [25].

To create a model using a neural network, it is necessary to designate an architecture for our neural network. The key points related to the architecture of neural networks are mainly divided into four parameters, namely the input layer (can be featured vector more than 1D), hidden layer (more than 1), output layer, and the number of neurons to connected layers. Most neural networks are fully connected, which means that each hidden layer can be connected to either its left or right side. The connection between layers is provided by several values called weights, which can be negative or positive and are initialized by a specific function in the programming space. The higher the value of weight, the greater the effects of layers on one another. Hence, initializing the optimal weights is the most important part of designing a model such that they minimize the prediction error. Gradient descent, backpropagation algorithms, and meta-heuristic methods are commonly used approaches for calculating optimal weights in neural networks. In this study, the process of designing optimal models using the neural network to recognize satisfactory models was performed using the grid search strategy in the TensorFlow library, such that the number of inputs, hidden layers, activation function, and optimization methods were tuned for the model.

### 3.4. Evaluation Criteria

To quantitatively validate the performance of the models, three statistical criteria of $R^{2}$ and Root Mean Square Error (RMSE) between the estimated and observed soil moisture, percent bias (PBIAS) and Bayesian Information Criterion (BIC) were used [26,27]. These three criteria are formulated below:

(6) $$R^{2}={\left[\frac{1}{N}\frac{\sum_{i=1}^{N}\left[\left(P_{i}-\underline{P}\right)\left(Q_{i}-\underline{Q}\right)\right]}{\sigma_{p}\sigma_{o}}\right]}^{2}$$

(7) $$\mathrm{RMSE}={\left(\frac{1}{N}\overset{N}{\underset{i=1}{\sum }}{\left[P_{i}-Q_{i}\right]}^{2}\right)}^{1/2}$$

(8) $$\mathrm{PBIAS}=\left(\frac{\;\left(\sum_{i=1}^{n}\left(P_{i}-Q_{i}\right)\right)}{\sum_{i=1}^{n}Q_{i}}\right)*100$$

(9) $$\mathrm{BIC}=k\times \ln\left(n\right)-2\times \ln\left(\hat{L}\right)$$

In the equations used, *N* denotes the total number of observations, $Q_{i}$ represents the observed values, $P_{i}$ represents the predicted values, *Q* represents the average of the observed values, and *P* represents the average of the predicted values. Additionally, $\sigma_{o}$ and $\sigma_{p}$ are the standard deviations of the observed and predicted values, respectively. In the BIC formula, *k* stands for the number of parameters, *n* is the count of observations, and $\hat{L}$ is the maximum likelihood estimate. Conceptually, higher values of $R^{2}$ (approaching 1) and lower values of RMSE (approaching zero) signify more desirable and effective model performance. However, it is crucial to note that the performance and estimation results of a model are heavily influenced by the specific samples selected for training. Thus, the metrics of $R^{2}$ and RMSE can vary when training different subsets of the dataset. The BIC facilitates optimal model selection by penalizing models with more parameters, thereby balancing the trade-off between fit and complexity. A lower BIC value suggests a more favorable balance, making it a useful metric for comparing models that differ significantly in parameter count. The percent bias (PBIAS) measures the average tendency of the simulated estimates to be larger or smaller than their corresponding observations. A PBIAS of zero indicates perfect agreement, with positive values showing an underestimation and negative values an overestimation by the model. This statistic is vital for determining if there is systematic bias in the model, which is crucial for model calibration and improvement. To ensure thorough validation, a K-fold (*k* = 5) cross-validation method was implemented, as it is strongly recommended for deploying machine learning techniques effectively.

## 4. Results and Discussion

### 4.1. The PC Analysis

**Figure 3:**
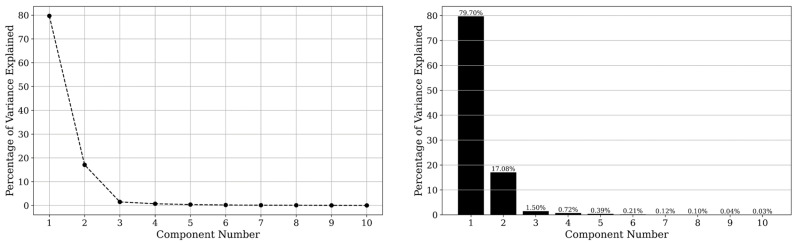
The scree plot (**left**) and the bar plot (**right**) of variance percentage in each PC.

**Figure 4:**
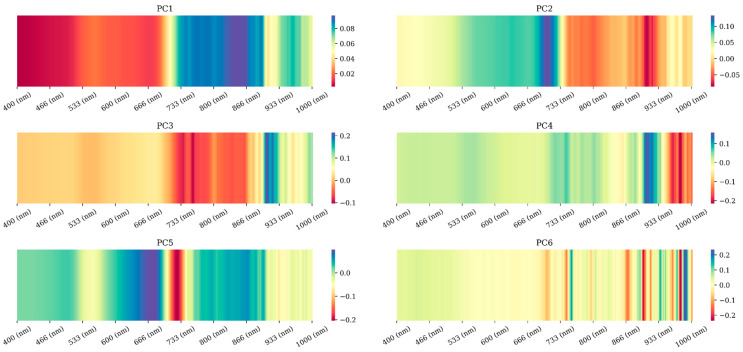
The contribution of each spectral band in the first 6 PCs.

Upon applying PCA to the hyperspectral images, our analysis shows a compelling narrative of data dimensionality and spectral feature representation. The scree plot (Figure 3) illustrates that the first two principal components are the most significant, explaining most of the variance within the dataset. This initial finding provided a rationale for focusing on these components for further model training and analysis. However, we decided to consider the first six PCs in the model training process, as minor spectral variations, corresponding with the water stress and structural variation in the crops, appeared in the higher components of PCA transformation. Figure 4 (loadings of spectral bands in each PC) proves this assumption that PC3, PC4, PC5, and PC6 contain minor spectral variation of specific wavelengths, regarding their coefficient of loading in the PCs.

**Figure 5:**
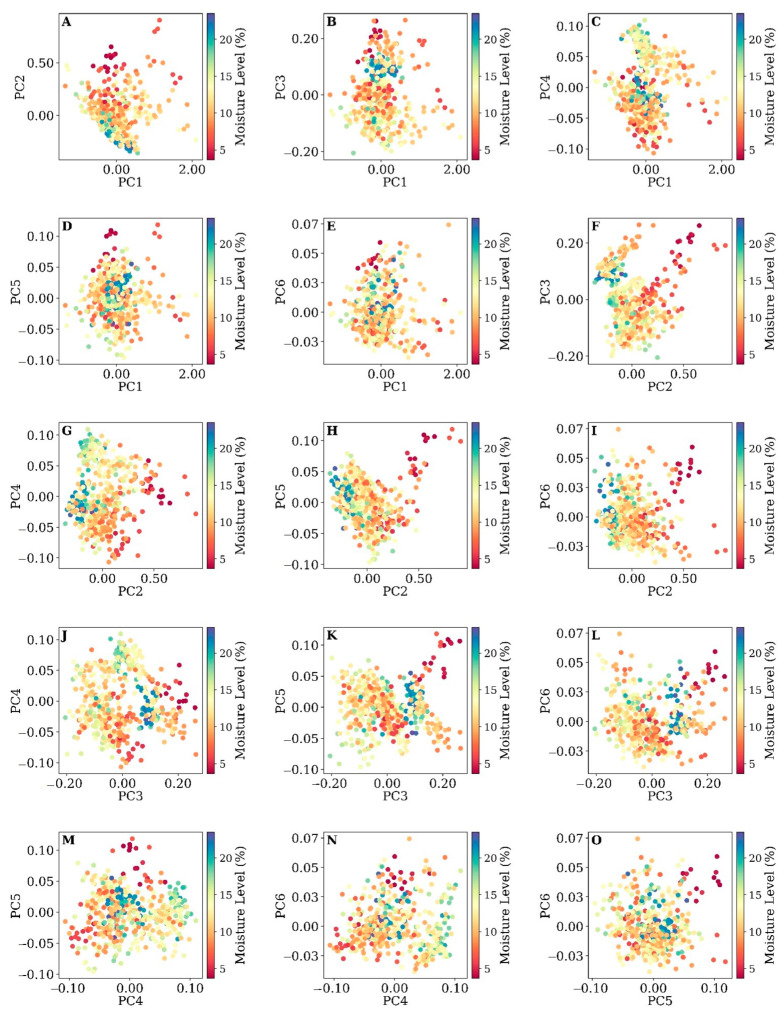
The distribution of soil moisture at a 10 cm depth in the 2D scatterplots of each pair of PCs (subplots **A**–**O**).

**Figure 6:**
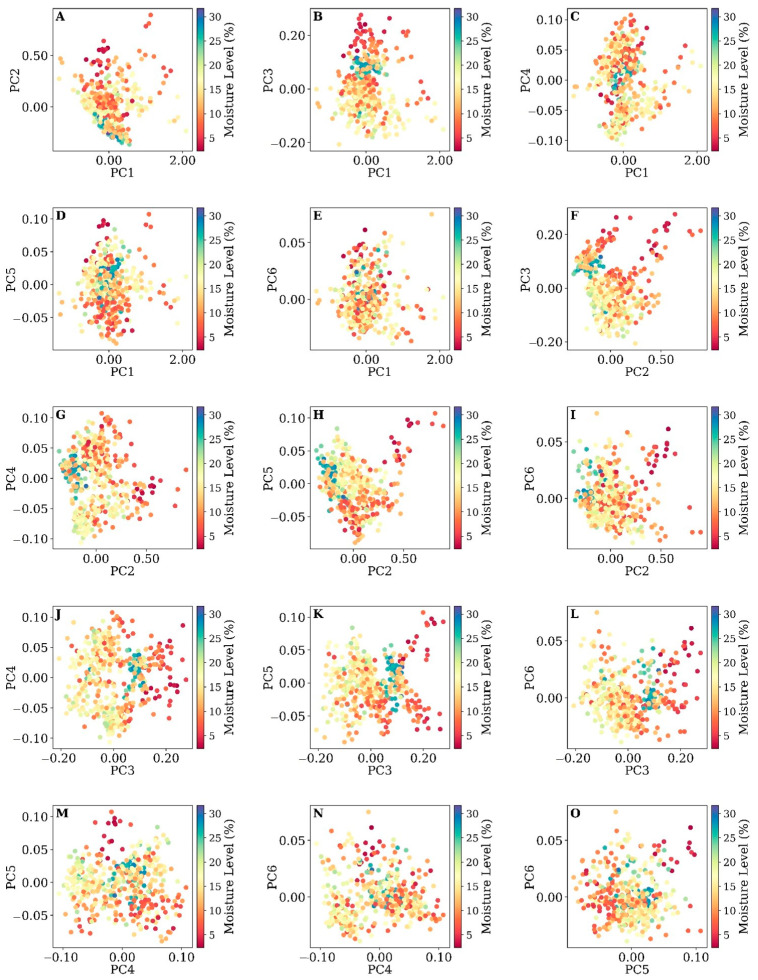
The distribution of soil moisture at 30 cm in the 2D scatterplots of each pair of PCs (subplots **A**–**O**).

To analyze the relationship between the first six PCs and soil moisture levels, we created scatterplots, each with two combinations of PCs, to see how the moisture samples are distributed and discriminated given their moisture levels. The goal of this analysis was to identify any meaningful spectral characteristics that could be linked to the canopy’s spectral properties, influenced by both the plants’ physiological state and the underlying soil moisture. Figure 5 and Figure 6 show the distribution of soil moisture samples at two depths: 10 cm and 30 cm, respectively, in a 2D scatterplot created based on the first six PCs.

Before moving forward to the scatterplots, each PC should be explained in terms of the wavelengths contributed, given the loading plots presented in Figure 4. In Figure 4, PC1 represents the overall brightness of the reflectance, which in the case of plant canopies, is influenced by the general health and vigor of the vegetation. While this component might be less directly related to soil moisture, healthier plants generally correspond to better-watered conditions, which might indirectly reflect the overall reflectance.

Some wavelengths have a significant contribution to PC2. PC2’s alternating loading patterns are indicative of the pigmentation differences within the plant canopy (regarding the significant contribution of wavelengths in the range of 645–730 nm and 800–950 cm [28,29]), which is related to variations in chlorophyll content. Many studies have reported that healthier plants with more available water display different reflectance characteristics than those under water stress [30,31,32]. This variation may have a relationship with soil moisture at the root level, so we expect PC2 to be a significant variable in our model.

In PC3 and PC4, almost single spectral bands or narrow regions of wavelengths (900 nm in PC3 and 950–1000 nm in PC4) with significant contributions are shown. These wavelengths from the NIR region of hyperspectral images were already used by previous studies for generating spectral indices related to plant structure [33]. Therefore, PC3 and PC4 might capture more useful information related to the vegetation’s structure, such as leaf area index (LAI) or canopy density, both of which can be correlated with soil moisture. This finding was highlighted in ref. [34], where they could find the variation of spectral indices related to plant structure with different levels of water stress. As a result, changes in leaf water content led to subtle shifts in reflectance in the NIR region, which could be picked up by these components.

PC5 and PC6, with their fine-scale oscillatory patterns, might detect very specific responses of the plant canopy to variations in soil moisture, including stress responses that only affect certain narrow bands of the spectrum. For example, these PCs might highlight the slight spectral shifts that occur when plants begin to experience water stress before more visible symptoms appear. Because of the existence of these specific wavelengths in PC5 and PC6, we decided to maintain these PCs in our model training process even though these PCs contain less than 1% of the variation.

As mentioned, to analyze the relationship between the soil moisture levels and spectral info, we not only created the 2D scatterplot of PCs but also the 2D scatterplot of soil moisture levels and some significant wavelengths with higher contributions in the PCs.

The distribution of soil moisture at a 10 cm depth (Figure 5), as interpreted through the PCA of plant canopy reflectance hyperspectral data, presents a nuanced understanding of the relationship between spectral characteristics and soil moisture levels. In this study, we observed that PC1 exhibits a significant gradient when plotted against the other PCs in terms of causing higher variation, suggesting higher discrimination of moisture samples in different levels (three clusters of low moisture content, mid-level, and high-level moisture content), suggestive of its overarching role in encompassing general reflectance related to canopy characteristics potentially influenced by soil moisture content. This relationship is particularly evident in the scatterplots of PC1 versus PCs 2 and 3, where systematic variance in soil moisture content along the PC1 axis was noted. The previous loading plots bolster this observation, as PC1’s uniform high loading across all wavelengths corresponded to general spectral intensity, which, in the context of canopy reflectance, could be influenced by the moisture conditions of the surface soil.

Conversely, PC2, when compared with PCs 3 through 6, presents a more heterogenous relationship with soil moisture content, indicating that PC2 might be capturing contrast variations in the canopy related to differential water stress levels. This aligns with the oscillatory loadings observed in PC2 from the loading plots, reflecting the contrast in reflectance across different wavelengths. Plots F through I of PC2 against the higher PCs show a complex and less-direct correspondence with soil moisture levels, suggesting the influence of additional canopy stress factors or other environmental variables. The inter-relationships among PCs 3 through 5 (Plots J, K, L, M, N) display a diffuse distribution of soil moisture content, potentially capturing more subtle or specific canopy and soil interactions, which are not linearly related to soil moisture at the investigated depth. The connection between these higher-order PCs and soil moisture content is less apparent in the scatterplots, which may imply that while they capture fine spectral details as indicated by the previous loading plots, these details might not be directly correlated with the soil moisture at the depth of 10 cm.

At a depth of 30 cm, the PC scatterplots (Figure 6) exhibit a meaningful relationship with soil moisture levels, indicative of the intricate interactions between canopy reflectance and subsurface water content. PC1’s influence appears attenuated in plots A through E, with soil moisture distribution showing a gradient along PC1’s axis, though less distinct than at 10 cm depth. This suggests a dilution of the direct linkage between surface reflectance and soil moisture as the depth increases, hinting at the canopy’s integration of long-term soil moisture availability rather than immediate variations at this greater soil depth.

In the PC2 scatterplots (Figure 6F–I), the distribution of soil moisture is notably scattered, lacking the clearer associations observed at shallower depths. Such a pattern may reflect the plant canopy’s composite response to environmental stressors, which includes but is not directly indicative of the moisture at a 30 cm depth. The lack of a strong, direct correlation might also point toward the canopy’s physiological adaptations and structural characteristics, which can obscure the spectral signals of deeper soil moisture contents. Comparing these results with the 10 cm depth plots, it becomes clear that as we probe deeper into the soil profile, the direct relationships captured by the hyperspectral canopy reflectance data become increasingly unclear. This trend underscores the complexity of the subsurface soil moisture dynamics and their expression through the plant canopy at various depths.

### 4.2. Crop Canopy Spectrum Analysis

In this section, we present the analysis of the interplay between select wavelengths, represented by key spectral bands, and the soil moisture at various depths within the cornfield. The wavelengths chosen for this investigation are those identified through PCA as having a significant influence (or causing higher discrimination of soil moisture levels) on the dataset, as indicated by their contribution to the loading plots. There are two rationales behind this aspect of our analysis. Firstly, we aimed to analyze how the relationship between soil moisture and canopy reflectance fluctuates across different soil depths as the plants progress through their growth season. This comparison helps us to understand how the depth of root water uptake influences the spectral signature of the canopy. Secondly, by examining the data from multiple dates, we sought to determine when and at what depth the correlation between soil moisture levels and canopy reflectance is strongest. Identifying these key times and depths allows us to pinpoint the most informative spectral bands for monitoring soil moisture, which can be critical for precision agriculture practices and water management strategies. Hence, given the previous studies conducted on corn’s growth stages, which are assigned the designations of VE to R6, from seeding to harvesting, we could assign each dataset to a specific growth stage. 15 June was placed in the V6 to V10 stages of plant growth and the data collection on 30 June was conducted at the V10 stage. The data collected on 11 July and 20 July included the stage of VT and R1, and the data collection conducted on 2 August and 14 August were at the stage of R1. Regarding the changes in root density and distribution over these stages of plant growth, we decided to compare the changes in the relationship between the soil moisture samples and canopy reflectance over the growth period to examine the effects of root structure changes on the relationship.

**Figure 7:**
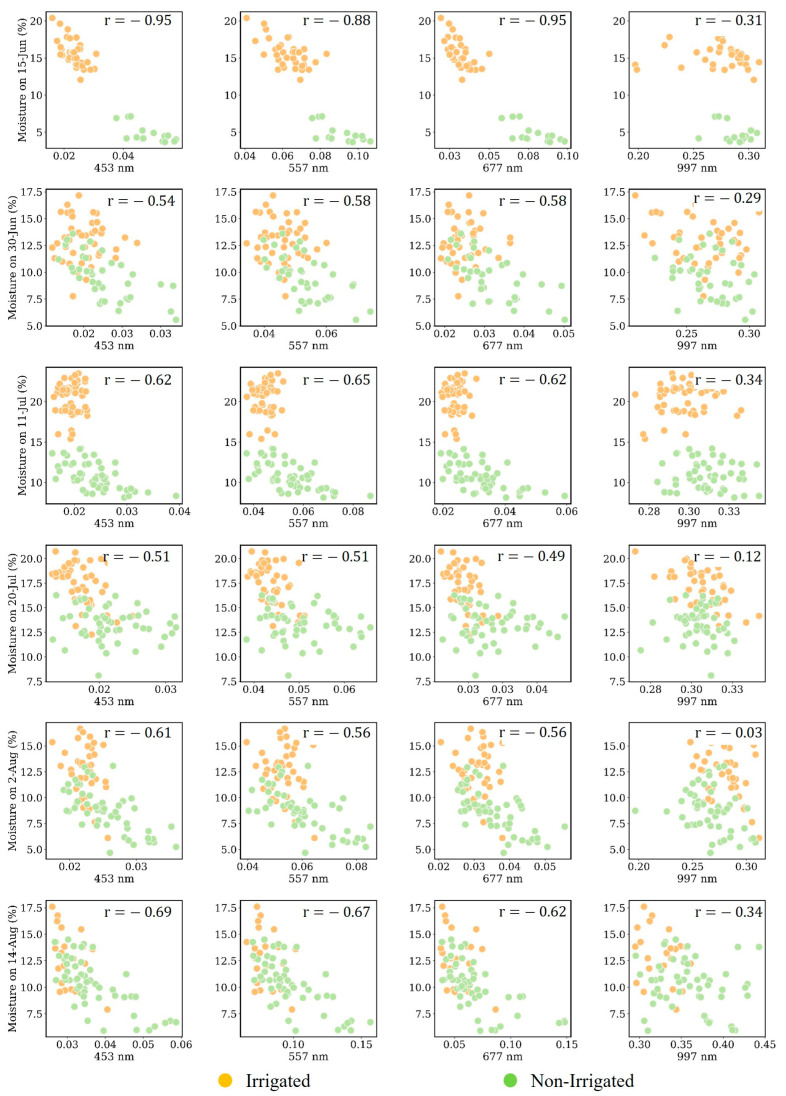
Pearson correlations between spectral bands and soil moisture at a 10 cm depth on different dates.

**Figure 8:**
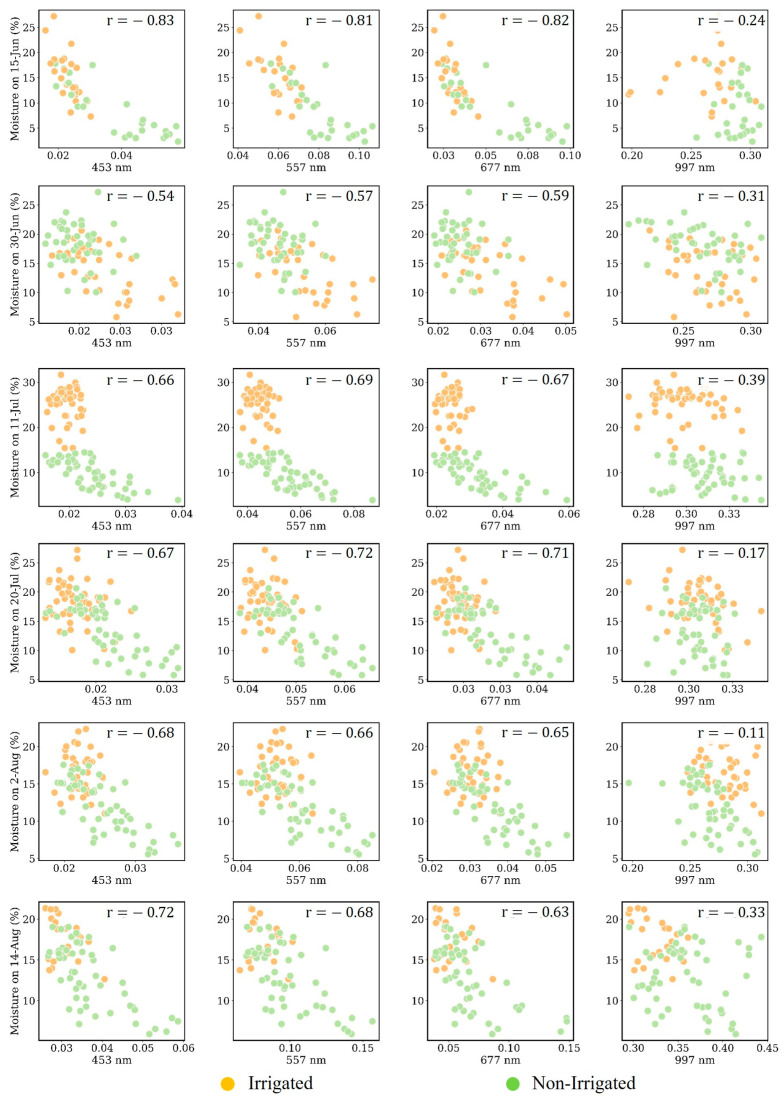
Pearson correlation values between spectral bands and soil moisture at a 30 cm depth on different dates.

We plotted the soil moisture data collected on various dates against the corresponding reflectance values to create a series of 2D scatterplots. Figure 7 and Figure 8 display these relationships for soil moisture depths of 10 cm and 30 cm, respectively.

From the figures, different depths show unique correlation profiles with the spectral data. Higher correlations at specific depths point to where the crop’s roots are most active in water absorption. For example, if shallower depths (e.g., 10 cm) demonstrate higher correlations early in the season, this is due to shallower root systems (Figure 7). There are two other reasons that we could see a higher correlation in the moisture samples collected on 15 June. The first reason is that, on this day, there was a very high difference between the moisture samples over the irrigated and non-irrigated plots, where the non-irrigated plots were under high water stress. At this stage of the corn growth stage (named V7 to V10), a critical stage for corn, any variation in available moisture can lead to significant physiological changes. These changes are readily detectable as alterations in the canopy reflectance, explaining the higher correlation when plants are undergoing such rapid development. This high water stress over the non-irrigated plots significantly affects the canopy spectrum and the crop’s leaf greenness, where we could even see the canopy color difference from simple RGB images. The correlation value of 0.95 in 453 nm and 677 nm (exactly the blue and red bands of the sensor) could show a higher correlation. This finding was also highlighted by Tucker in 1979, who used a red band for vegetation monitoring [35].

There are two other findings from the correlation values in Figure 7. The first finding is that there is a high correlation between soil moisture measured from irrigated and non-irrigated plots at a depth of 10 cm and spectral information in visible bands. Higher correlation at the specific depth of 10 cm in the moisture samples collected on 15 June proves that over the V6 to V10 stages of plant growth, the root system is highly active in the upper soil layers during these vegetative stages, with water uptake being vigorous to support rapid growth. This high activity in the root zone means that changes in soil moisture at this depth can significantly impact the plant’s physiological status, including its canopy reflectance, especially in the bands sensitive to chlorophyll and water content. This finding was also highlighted by Jackson et al. [36]. Canopy responsiveness is another specific reason, proving the higher correlation between samples at a depth of 10 cm and canopy reflectance values. The canopy is particularly responsive to water status during early growth stages (V7 to V10, 15 June). Soil moisture at a 10 cm depth directly influences the health and vigor of the canopy, affecting its spectral reflectance properties. This sensitivity is especially evident in bands that capture the chlorophyll content (Figure 7, and NIR and red-edge wavelengths show a higher relation with the soil moisture samples at 10 cm). This finding was mentioned by Gitelson et al. in 2003, where they could highlight a relationship between leaf chlorophyll content and spectral reflectance in the red edge and near-infrared regions to create an algorithm for non-destructive chlorophyll assessment in higher plant leaves [37].

The correlation values between moisture samples and the crop’s canopy spectrum information decreased for moisture samples measured on 30 June. On this date, although there was a reasonable difference in soil moisture content in the samples over the irrigated and non-irrigated plots, the crop’s canopies were similar at their highest levels of greenness and chlorophyll content. This date could be considered as the mid-vegetative stage of plant growth or mid-stage of the growing season when the plant usually has higher levels of chlorophyll [38] as well as a higher resiliency to environmental stress (high air temperature or low water available) [39]. Therefore, the higher difference in soil moisture sample over the irrigated and non-irrigated plots could not cause significant correlation (or variation in canopy spectrum), and the drop in the correlation values across all the spectral variables is expected.

As the crop matures and roots grow deeper, we expect to see an increase in correlation at greater depths (30 cm), specifically over the non-irrigated plots where the root starts going down to access water [40]. As displayed in Figure 8, the higher correlation occurred at the depths of 30 cm between 11 July and 20 July, which is the growing season. Over these two data collection dates, there were no rainy days, the crops in non-irrigated plots were under high water stress, and irrigated plots were irrigated regularly. This caused a situation where there was not only a high difference in soil moisture samples over the irrigated and non-irrigated plots, but the crop’s canopy spectrum was also significantly different over irrigated and non-irrigated plots. The higher correlation between soil moisture samples and the crop’s canopy spectrum can also be associated with the corn’s root functioning at this stage. This stage is also called the tasseling stage (VT), when just before and during tasseling, the corn plant is still typically at its peak greenness, indicating high chlorophyll concentration as the plant prepares to reproduce [41,42]. At this stage, the crop root is functioning critically in various terms, such as water uptake, where the plant roots are actively up taking water to support the high transpiration demands of the large leaf area. Adequate water is crucial at this stage, as it directly impacts tassel development and the success of pollination. Thus, since there has not been adequate water in non-irrigated plots, we expected a high spectrum variation due to the root–canopy response and eventually a higher relationship between soil moisture levels and canopy spectrum. The non-irrigated plot moisture levels revealed a high correlation between moisture levels at 30 cm and the canopy spectrum (the scatterplots of samples collected on 11 July and 20 July in Figure 8).

Root growth and soil exploration are two other factors that we considered while analyzing the moisture samples measured on 11 July and 20 July. On these two dates, because of the higher water stress of the plant and low levels of soil moisture, the corn roots began exploring the soil and going deeper to access water [43,44], potentially making the root system more extensive at a 30 cm depth, causing higher correlation values at this depth.

During the R1 to R6 stages of corn growth, which encompass the reproductive phase from silking to physiological maturity [45], we collected data twice, on 2 August and 14 August. The functioning of the roots in irrigated and non-irrigated plots can reveal several insights. The first noticeable result is that the correlation values between the measured soil moisture at 30 cm and reflectance in visible wavelengths with a value between 0.60 to 0.65 during July remained unchanged or slightly increased to a value of between 0.65 and 0.73 in moisture samples collected at a 30 cm depth measured on 2 August and 14 August (Figure 8). This finding proves that, in August, the plant’s roots at a lower depth (30 cm) become more functionally active, and moisture variation causes higher variation in canopy spectrum information.

Another finding from the samples measured over this period is that we can see a better relationship between the moisture samples of non-irrigated plots at a depth of 10 cm compared to the samples measured during July. This is due to the non-irrigated plants’ heightened sensitivity to changes in water availability, where a slight increase in moisture leads (resulting from rainfall) to a significant spectral response. This sensitivity is a result of the plants being more attuned to water stress, making their canopies more responsive to fluctuations in water supply.

### 4.3. Evaluation of Learning Tools

For the SVR, we explored several kernel types including RBF, linear, and polynomial to identify the best fit for our data characteristics. The regularization parameter C was varied across a broad spectrum (0, 10, 50, 100, 200) to determine the optimal balance between model complexity and training accuracy. The epsilon values (0.1, 0.2, 0.5), controlling the width of the margin of tolerance where no penalty is given to errors, and gamma values (0.01, 0.001, 0.002), which define the influence of a single training example, were carefully adjusted to fine-tune the model’s sensitivity to the training data.

For the RF, adjustments were made to the maximum depth of the trees (3, 5, 7, 9, 20) to prevent overfitting while ensuring sufficient model depth for capturing data complexities. The number of estimators was also varied (20, 50, 100, 200) to find an optimal number that provides the best generalization performance without becoming computationally prohibitive.

In the case of the GBM, we tested different configurations by altering the number of estimators (100, 200, 300) and the learning rate (0.02, 0.05, 0.1, 0.2) to control the training speed and overfitting potential. The subsample parameter was set at 0.5 and 0.6 to introduce stochasticity in the learning process, thereby improving the robustness of the model against noise in the training data.

For the Artificial Neural Network (ANN), the architecture was optimized by varying the number of neurons per layer (32, 40, 50, 64) and experimenting with different optimizers (Adam, SGD) along with a range of learning rates (0.1, 0.01, 0.002, 0.0005). The batch sizes (32, 64, 100, 256) were adjusted to optimize computational efficiency and convergence speed during training. This allowed the ANN to effectively learn complex patterns and interactions within the dataset. The process of model training was conducted with 70% of the total samples (548 samples), and the remaining samples were used for evaluation of the models.

The SVR model was configured with a radial basis function (RBF) kernel, regularization parameter C = 100, loss tolerance = 0.2, and kernel coefficient γ = 0.01 to effectively manage the trade-off between model complexity and learning accuracy from the data. For the RF regressor, we selected an ensemble of 50 trees with a maximum depth of 9, employing bootstrap samples, ensuring robust random sampling, and preventing overfitting.

Further, our GBM was tuned with parameters set for optimal depth and node creation using a learning rate of 0.2 and 100 estimators. The ANN, designed using the TensorFlow framework, comprised multiple dense layers with 40 neurons each, ReLU activation, and dropout of 0.3 to prevent overfitting, which was crucial for learning non-linear relationships in the data. An Adam optimizer with a learning rate of 0.0005 was utilized to minimize the mean squared error across 400 epochs, demonstrating the model’s efficiency in learning from the training data while validating against the test set.

The assessment of machine learning models for estimating soil moisture at a depth of 10 cm revealed significant differences in performance. Presented in Table 2, the models evaluated included RF, GBM, SVM, and ANN. The ANN emerged as the most accurate model, recording an R^2^ of 0.72, indicating that it could explain 75% of the variance in soil moisture at this depth. The performance of the ANN is attributed to its nature and power in capturing the complex nonlinear relationships inherent in the soil moisture variables compared to other algorithms [46].

Additionally, it achieved the lowest RMSE of 2.30%. The SVM followed with an R^2^ of 0.68, while GBM and RF had lower but still substantial R^2^ values of 0.67 and 0.63, respectively. The progression from RF to ANN highlighted a clear trend: as the complexity of the models increased, so did their ability to capture and predict the nuances of soil moisture variability. In terms of Pbias error, the ANN model had the best performance at a 10 cm depth, with a Pbias of 0.055%, indicating the least amount of bias and highly accurate predictions with minimal over or underestimation. The SVM model followed closely, with a Pbias of −0.32%, demonstrating only a slight tendency toward underestimation. The GBM model had a Pbias of 2.41%, reflecting a moderate level of bias but still maintaining a good balance between prediction accuracy and systematic error. The RF model exhibited the highest Pbias at 2.66%, suggesting a greater degree of bias and a higher likelihood of over- or underpredicting soil moisture.

Further statistical analysis supports the superior performance of the ANN model (Table 3). The ANN achieved an F-statistic value of 432.7, indicating a highly significant model fit with a practically negligible probability of F-statistic (*p* < 6.06 × 10^−46^). This was complemented by the lowest BIC score of 677.5 among the models, suggesting that despite its complexity, the ANN model offers a desirable balance of fit and simplicity. The *t*-tests conducted on the slopes and intercepts of the regression models reinforce this conclusion, with the ANN model showing a statistically significant *t*-test/slope value of 20.87 and a near-zero *t*-test/intercept value, suggesting minimal deviation from the origin. These statistical validations cement the ANN model’s status as a robust and reliable method for soil moisture estimation, marking it a preferable choice for applications that require high accuracy such as precision agriculture and water resource management.

**Figure 9:**
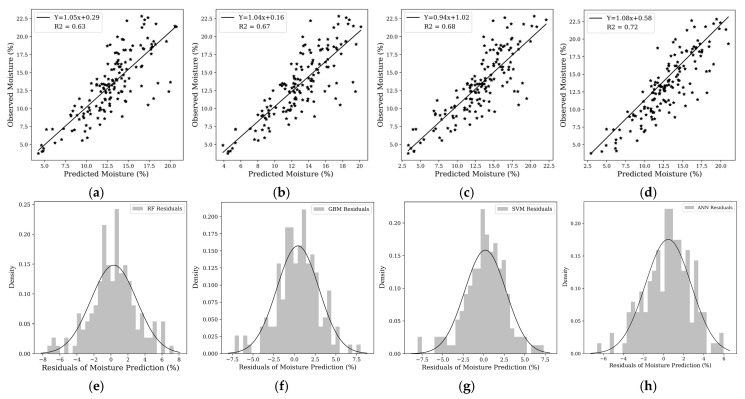
(**a**–**d**) A 1:1 line of observed and predicted soil moisture of 164 test samples (30% of the data) at 10 cm depth, obtained from the RF (Random Forest), GBM (Gradient Boosting Model), SVM (Support Vector Machine), and ANN (Artificial Neural Network), respectively. (**e**–**h**) show the corresponding histograms of residuals obtained from the algorithms.

Figure 9 shows that the 1:1-line scatterplots and the distribution of residuals resulted from the machine learning algorithms. Figure 9a through Figure 9d depict 1:1-line scatterplots of observed versus predicted soil moisture for each algorithm. Consistent with the previously reported statistical metrics, the ANN model (Figure 9d) demonstrated the closest adherence to the 1:1 line, reflected in an R^2^ value of 0.72, indicating a strong positive correlation between predicted and observed values. This is corroborated by the narrow spread around the line of unity, suggesting accurate predictions with minimal bias. The SVM model (Figure 9c) also showed tight clustering near the 1:1 line, albeit with slightly more dispersion than the ANN, corresponding to its R^2^ of 0.68. The RF and GBM models (Figure 9a,b), while still showing a positive correlation with R^2^ values of 0.60 and 0.63, respectively, exhibit a broader scatter of points, implying less-accurate prediction of soil moisture levels.

Figure 9e–h provide insight into the distribution of residuals for each model’s moisture predictions. A residual is the difference between the observed value and the model’s prediction, and an ideal model would have a residual distribution that is narrowly centered around zero. The ANN model (Figure 9h) achieves a nearly symmetrical distribution of residuals with a sharp peak around zero, indicating that most of its predictions were very close to the actual values. The SVM model (Figure 9g) displays a similar pattern, with a slightly wider spread, which aligns with its marginally lower R^2^ value. In contrast, the RF and GBM models (Figure 9e,f) show wider distributions, reflecting the greater variability in their prediction accuracy. The histograms reaffirm the superior performance of the ANN and SVM models over the RF and GBM models for soil moisture estimation at this depth.

**Figure 10:**
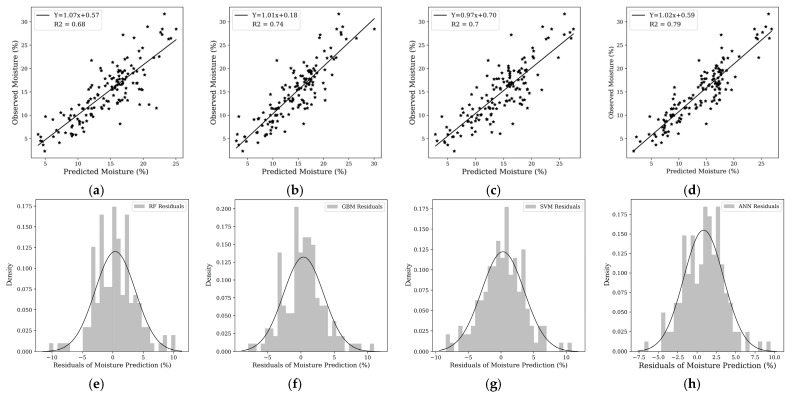
(**a**–**d**) A 1:1 line of observed and predicted soil moisture of 164 test samples (30% of the data) at a 30 cm depth, obtained using RF (Random Forest), GBM (Gradient Boosting Model), SVM (Support Vector Machine), and ANN (Artificial Neural Network), respectively. (**e**–**h**) show the corresponding histograms of the residuals obtained from the algorithms.

At a depth of 30 cm, all of the models demonstrated enhanced performance, with the ANN leading (R^2^ = 0.79, Table 4) and the GBM not far behind (R^2^ = 0.74, Table 4). The SVM also displayed a commendable R^2^ of 0.70, showcasing its robustness in modeling at this depth. The scatterplots corroborate these findings, with a tighter cluster of points around the 1:1 line for GBM and SVM (Figure 10b,c), and the residual plots revealed a relatively narrow spread for these models (Figure 10f,g), suggesting a higher consistency in predictions. In terms of biases errors, the ANN model showed the best performance in terms of Pbias, with a value of 1.19%, indicating minimal bias and highly reliable predictions without significant over- or underestimation. The GBM model also performed well, with a Pbias of 2.12%, reflecting a reasonable balance between accuracy and bias. The Random Forest (RF) model had a slightly higher Pbias of 2.34%, indicating a modest increase in systematic bias. In contrast, the SVM model had the highest Pbias at 2.74%, making it the most prone to over- or underpredicting soil moisture.

The further statistical tests presented in Table 5 also prove the better performance of the ANN model. In this table, the ANN model shows the highest F-statistic of 640.1, indicating the strongest relationship between predicted and observed values and the greatest statistical significance. This is supported by the probability (Prob) of the F-statistic, where the ANN model has the lowest value at 2.01 × 10^−55^, which effectively demonstrates a virtually zero chance that the model’s predictive capabilities are due to random variation. The *t*-test/slope values are all significant for the models, with the ANN model exhibiting the highest value of 25.29, suggesting a strong linear relationship between the predicted and observed soil moisture levels. The ANN’s *t*-test/intercept is closest to zero at 0.98, showing minimal bias in its predictions. Conversely, the RF model exhibits a negative intercept, suggesting a consistent underestimation of the soil moisture content.

**Figure 11:**
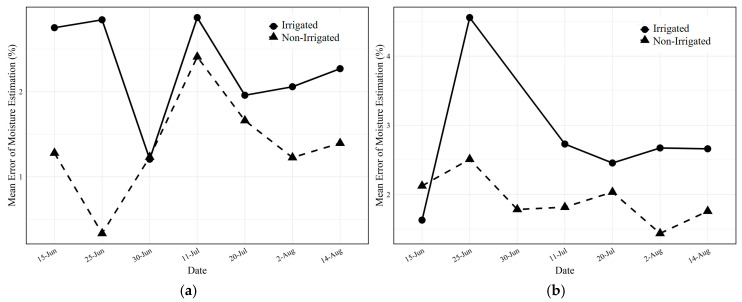
The breakdown of the estimation error resulting from the trained ANN model at (**a**) 10 cm and (**b**) 30 cm depths.

Figure 11 presents the error breakdown of soil moisture estimates at 10 cm and 30 cm soil depths, determined using the ANN model over several dates.

Figure 11 shows that, generally, non-irrigated plots yielded more accurate soil moisture estimates than irrigated ones across most dates (less than 2% at 10 cm and less than 3% at 30 cm). As previously examined, the early growth stage on 15 June is characterized by minimal root activity at 30 cm, which may lead to a lesser correspondence with canopy spectral data and, consequently, increased estimation errors. Additionally, the substantial estimation errors across various depths on 25 June can be attributed to the preceding rainfall, which likely led to homogenized soil moisture due to saturation, hampering the spectral differentiation typically seen in the canopy, a phenomenon referred to as spectral saturation. This effect aligns with the observations in the scatterplots (Figure 7 and Figure 8) and is consistent with the spectral responses expected during the early growth stages, where even minor shifts in water content can significantly alter canopy spectra.

Figure 11b indicates that the smallest relative errors for the non-irrigated plots occurred in the 30 cm depth samples collected on 11 and 20 July. During these dates, the plants in non-irrigated plots experienced moisture stress at shallower levels, prompting root systems to extend deeper in search of water. This stress response is reflected in the higher correlation (Figure 8), which inversely correlates with a lower RMSE, indicating enhanced model accuracy for non-irrigated samples in July.

The error breakdown for the July period in Figure 11b highlights intriguing results for the data collected on 2 and 14 August, corresponding to the R1 growth stage. Here, the correlation between soil moisture at a depth of 30 cm in non-irrigated plots and canopy spectral data was stronger than at shallower depths; a relationship likely influenced by the moisture-responsive root system at these lower depths, leading to improved estimation accuracy.

### 4.4. Comparison with Previous Studies

When comparing our findings with previous research, our study highlights the promising accuracy of advanced machine learning models, particularly ANNs, for soil moisture estimation across multiple depths. Notably, the ANN achieved an R^2^ of 0.72 at 10 cm, aligning closely with the high performance benchmarks set in other studies on soil moisture estimation in agricultural contexts. This reinforces the assertion that complex models like ANNs, capable of capturing nonlinear interactions, outperform simpler algorithms. For instance, as noted by Ding et al. [47], Nawar and Mouazen [48], RF consistently delivers robust predictions of soil properties, particularly in arid, heterogeneous soils. However, our findings indicate that while RF is robust (R^2^ of 0.63), the ANN’s accuracy surpasses it, suggesting that ANNs are better suited for capturing soil moisture variability in complex environments, as also observed by Chen et al. [49] and Lindner et al. [50].

The selection of critical spectral wavelengths in our study, particularly 453 nm, 557 nm, 677 nm, 814 nm, and 997 nm, directly contributes to the high accuracy of soil moisture models, especially for ANN and SVM. This is in line with Ge et al. [10], who found that pretreatment of spectral indices, particularly those emphasizing green, red, and red-edge wavelengths, enhanced model performance significantly. Also, Guan et al. highlighted the importance of reflectance data from red, green, blue, and NIR bands, which showed correlations with SWC measurements that were as strong as or stronger than many vegetation indices, suggesting that these bands should be incorporated into machine learning models [51]. Similarly, our study found that targeted spectral data enhanced the predictive capability of the ANN, achieving a low MAE of 1.83% at 10 cm, reflecting a robust accuracy consistent with other studies that leveraged pretreatment for SMC predictions. These findings suggest that carefully selected wavelengths amplify model precision, particularly when applied with advanced machine learning techniques.

At greater soil depths, our results mirror previous studies in highlighting the challenge of maintaining prediction accuracy as depth increases. Specifically, Zhu et al. [52] reported that models exhibit a drop in R^2^ with increasing depth due to reduced spectral signal quality from subsurface layers and increased canopy interference. In our study, model performance at a 30 cm depth showed a similar trend for all models except the ANN, consistent with the observations by Ge et al. and Zhu et al. of diminishing prediction accuracy for soil moisture in deeper layers. Additionally, Chen et al. [49] and Thevs et al. [53] indicated that the root-zone depth and vegetation canopy significantly impact accuracy, with models demonstrating heightened sensitivity to shallow soil moisture levels. Our results support these findings, showing that the ANN and SVM achieved better predictive performance at a 10 cm depth, where spectral data strongly correspond to moisture content due to shallow root uptake.

Interestingly, our study also echoes the insights from Chen et al. [49] regarding the anti-interference capabilities of RF in handling noise and outliers, which allows it to perform reliably under variable moisture conditions. While the RF in our study performed reasonably well, particularly at a 10 cm depth, the ANN’s superior generalization to complex soil interactions resulted in a higher accuracy, as reflected by the ANN’s F-statistic of 432.7. Moreover, Chen et al. [49] found that SVM generally performs well with complex datasets, though it is sensitive to noisy data. Similarly, our SVM model achieved a strong R^2^ of 0.68 at 10 cm, proving its efficacy in scenarios with limited noise but showing less resilience at 40 cm, where outliers impacted its accuracy more noticeably.

In line with Shidan Zhu et al. [54], who highlighted that the effectiveness of ML models in soil moisture estimation varies across root depths, our study confirms that the ANN consistently performs best across all depths, with the SVM and GBM following closely at shallower levels. Specifically, at 30 cm, the ANN reached an impressive R^2^ of 0.79, underscoring its capacity to manage soil moisture prediction even as depth increases; a challenge previously noted by Chen et al. [49] for models applied to varying soil depths. This result indicates that while depth inherently complicates moisture estimation, the ANN provides a reliable solution for applications where detailed, depth-sensitive moisture data are essential.

Overall, our findings resonate strongly with previous research, reaffirming that machine learning, and particularly ANNs, plays a crucial role in achieving high soil moisture prediction accuracy across variable depths. The comparable performance metrics, such as high R^2^ values and low error rates, support that advanced ML models, tailored with critical spectral information, are capable of capturing soil moisture intricacies across layers. This consistency with prior studies underscores ANN’s potential as a primary tool for precision agriculture and water management applications, especially when model selection is aligned with the target depth of moisture estimation.

### 4.5. Soil Moisture Maps

**Figure 12:**
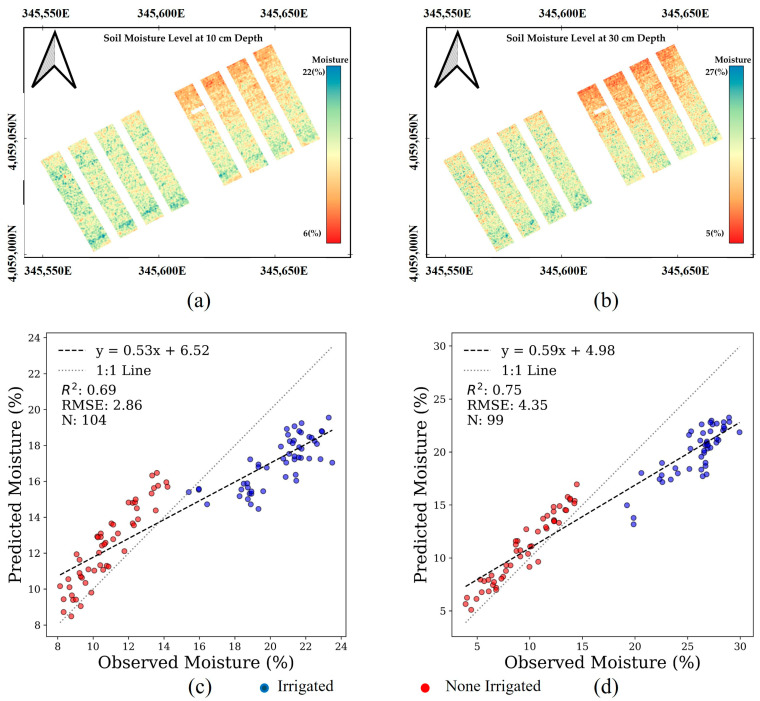
Soil moisture maps at depths of (**a**) 10 cm and (**b**) 30 cm, and the corresponding scatterplots plotted in (**c**,**d**) generated by the ANN model (N represents the number of samples).

In this section, we extend the application of the developed ANN model to analyze another dataset derived from hyperspectral data collected on another date using the same drone (this dataset has not been used in training or testing our developed ANN model). The analysis using the ANN model on an alternative hyperspectral dataset for soil moisture estimation revealed insightful patterns about the model’s adaptability and precision across both depths (Figure 12). The soil moisture maps generated by the ANN model distinctly illustrate the moisture levels at depths of 10 and 30 cm, successfully capturing the difference between irrigated and non-irrigated plots. These visualizations are particularly potent, as they show the model’s capability to delineate soil moisture variations spatially across the field, confirming its effectiveness in recognizing and reflecting the irrigation impacts at multiple sub-surface levels.

Further scrutiny based on the scatterplots in Figure 12c,d of observed versus estimated soil moisture highlights a consistent underestimation in irrigated plots at a shallower depth of 10 cm, which is attributed to the plant resistance during the growing season. The dataset used in the ANN model was captured on 31 July, when the irrigated plots had higher levels of soil moisture, and the non-irrigated plots were under water stress. In the spectral analysis section, we mentioned that spectral saturation phenomena over the canopy spectrum info of irrigated plots would not be able to provide information about the higher moisture levels. Thus, the canopy spectral-informed ANN model would not be able to accurately estimate moisture over soil with higher levels of moisture. However, the estimation of non-irrigated samples was remarkably accurate, maintaining a RMSE of around 3% across all depths, showcasing the model’s robustness in scenarios with less variation in water inputs. Interestingly, the accuracy of moisture estimates for both irrigated and non-irrigated plots improved significantly at a depth of 30 cm (Figure 12d).

### 4.6. Limitations and Suggestions for Future Works

In this study, while our methodologies have demonstrated considerable potential, they are accompanied by several limitations that could be addressed in future work. Firstly, the specificity of the crop type used in our experiments might limit the general applicability of our findings. Different crops have unique water uptake characteristics that influence soil moisture dynamics differently. By extending our research to include a variety of crops, we can test the robustness of our models and potentially increase their applicability to a broader range of agricultural contexts.

Secondly, our experiments were predominantly conducted in sandy soil, known for its distinct hydrological properties that can significantly affect soil moisture behavior. Exploring the efficacy of our methods across different soil types such as clay or loam could offer insights into how soil texture influences the performance of our models. This expansion would not only validate the effectiveness of our approaches in various settings but also enhance the precision of moisture estimates in soils with different retention capacities and textural properties.

A significant limitation encountered during our study was the issue of spectral saturation in samples with higher moisture levels, which predominantly led to underestimations in our predictions. This observation suggests an opportunity to refine the loss function in the ANN model. By adjusting the loss function to penalize underestimation errors more significantly, the model could be tuned to address this bias, improving accuracy in high moisture scenarios. Such an approach would make the model more reliable and robust, particularly in environments where soil moisture reaches higher levels.

Furthermore, incorporating additional variables such as plant structure and growth stage could significantly enhance model performance. These factors play crucial roles in the soil–plant–atmosphere continuum and could provide valuable predictors of soil moisture. Integrating these variables as categorical inputs in our models could help capture the dynamic interactions between plant growth stages and soil moisture, offering a more nuanced and accurate prediction model. This adjustment could lead to better-informed irrigation decisions, optimized for the specific growth phases of crops, ultimately contributing to more sustainable agricultural practices.

## 5. Conclusions

In this study, the integration of a drone-mounted hyperspectral sensor (L-Pika) and machine learning algorithms has been leveraged to accurately assess soil moisture at different depths within vegetated corn fields. By employing PCA and ANNs, this research has sought to determine whether such methods could reliably estimate soil moisture. The findings demonstrated PCA’s effectiveness in identifying significant variables of PCs, providing discrimination of soil moisture levels and the relationship between PCs and soil moisture levels, into an ANN model for soil moisture estimation. The finding indicated the superior performance of the trained ANN model over other machine learning algorithms, given the numerical values of the evaluation criteria. The numerical evaluation of the model’s performance showed that the ANN model could estimate the soil moisture at a depth of 30 cm over the non-irrigated or high-water-stress condition.

However, the error analysis showed that, under specific conditions caused by environmental factors such as rainfall and air temperature variation, a weaker relationship between soil moisture levels and canopy spectral information could be seen, resulting in a higher estimation error. Addressing these limitations, future research could integrate additional variables, such as thermal imagery and land surface temperature, to develop a more generalized model with wider applicability.

This study was driven by a set of main objectives and structured based on pivotal research questions. One of the primary aims was to unravel the relationship between root zone water content and canopy reflectance, interrogating the depths at which this relationship was most evident. In this context, the investigation successfully revealed that the correlation is indeed significant, particularly at the critical root-active depths that are central to plant health and water uptake. This finding underscores the potential of using spectral data as a reliable proxy for soil moisture content, especially at depths where root systems are actively engaging with the soil–water matrix. The analysis of canopy spectral information also showed that certain wavelengths, particularly those responsive to changes in chlorophyll content and water stress, were informative and significant in predicting soil moisture with high precision. These findings provide valuable directions for future enhancements in spectral analysis, enabling more accurate monitoring and management of soil moisture for optimal crop growth.

## Figures and Tables

**Table 1:** The flight parameters definable in the L-Pika sensor.

Sensor Parts	Details	
Spectral information	Spectral range	400–1100 (nm)
	Spectral channel	281
	Bandwidth	2.1 (nm)
	Resolution	2.7 (nm)
Spatial information	Width	900
	Length	1200
Lens info	FOV (degree)	17.6
	Frame rate	249 fps

**Table 2:** Models’ performance for test samples collected at a depth of 10 cm.

Model	R^2^	RMSE (%)	Pbias
RF	0.63	2.72	2.66
GBM	0.67	2.57	2.41
SVM	0.68	2.52	−0.32
ANN	0.72	2.30	0.055

**Table 3:** Models’ performance for test samples collected at a depth of 10 cm.

Model	F-Statistic	BIC	*t*-Test/Slope	*t*-Test/Intercept	Prob (F-Statistic)
RF	257.2	732.2	160.3	−0.33	3.52 × 10^−34^
GBM	308.5	714.3	17.56	−0.19	7.98 × 10^38^
SVM	318	711.2	17.83	1.42	1.09 × 10^−38^
ANN	432.7	677.5	20.87	−1.39	6.06 × 10^−46^

**Table 4:** Models’ performance for test samples collected at a depth of 30 cm.

Model	R^2^	RMSE (%)	Pbias
RF	0.68	3.42	2.34
GBM	0.74	3.04	2.12
SVM	0.70	3.27	2.74
ANN	0.79	2.71	1.19

**Table 5:** Models’ performance for test samples collected at a depth of 30 cm.

Model	F-Statistic	BIC	*t*-Test/Slope	*t*-Test/Intercept	Prob (F-Statistic)
RF	330.7	788.8	18.18	−0.69	1.89 × 10^−39^
GBM	425.8	761.7	20.63	0.24	2.91 × 10^−45^
SVM	342.1	785.3	18.495	0.86	3.34 × 10^−40^
ANN	640.1	714.4	25.29	0.98	2.01 × 10^−55^

## Data Availability

Upon request is available.

## References

[1] Chartzoulakis K., Bertaki M. (2015). Sustainable water management in agriculture under climate change. Agric. Agric. Sci. Procedia.

[2] Wang E., Smith C.J. (2004). Modelling the growth and water uptake function of plant root systems: A review. Aust. J. Agric. Res..

[3] Hopmans J.W., Bristow K.L. (2002). Current capabilities and future needs of root water and nutrient uptake modeling. Adv. Agron..

[4] Seneviratne S.I., Corti T., Davin E.L., Hirschi M., Jaeger E.B., Lehner I., Orlowsky B., Teuling A.J. (2010). Investigating soil moisture–climate interactions in a changing climate: A review. Earth Sci. Rev..

[5] Stafford J. (1988). Remote, non-contact and in-situ measurement of soil moisture content: A review. J. Agric. Eng. Res..

[6] Rasheed M.W., Tang J., Sarwar A., Shah S., Saddique N., Khan M.U., Imran Khan M., Nawaz S., Shamshiri R.R., Aziz M. (2022). Soil moisture measuring techniques and factors affecting the moisture dynamics: A comprehensive review. Sustainability.

[7] Jonard F. (2012). Soil Water Content Estimation Using Ground-Based Active and Passive Microwave Remote Sensing: Ground-Penetrating Radar and Radiometer. Ph.D. Thesis.

[8] Babaeian E., Sadeghi M., Jones S.B., Montzka C., Vereecken H., Tuller M. (2019). Ground, proximal, and satellite remote sensing of soil moisture. Rev. Geophys..

[9] Aboutalebi M., Allen L.N., Torres-Rua A.F., McKee M., Coopmans C., Thomasson J.A. Estimation of soil moisture at different soil levels using machine learning techniques and unmanned aerial vehicle (UAV) multispectral imagery. Proceedings of the Autonomous Air and Ground Sensing Systems for Agricultural Optimization and Phenotyping.

[10] Ge X., Ding J., Jin X., Wang J., Chen X., Li X., Liu J., Xie B. (2021). Estimating agricultural soil moisture content through UAV-based hyperspectral images in the arid region. Remote Sens..

[11] Zhang Y., Han W., Zhang H., Niu X., Shao G. (2023). Evaluating soil moisture content under maize coverage using UAV multimodal data by machine learning algorithms. J. Hydrol..

[12] Savitzky A., Golay M.J. (1964). Smoothing and differentiation of data by simplified least squares procedures. Anal. Chem..

[13] Abdi H., Williams L.J. (2010). Principal component analysis. Wiley Interdiscip. Rev. Comput. Stat..

[14] Smith L.I. (2002). A Tutorial on Principal Components Analysis.

[15] Jolliffe I.T. (2002). Principal Component Analysis for Special Types of Data.

[16] Cangelosi R., Goriely A. (2007). Component retention in principal component analysis with application to cDNA microarray data. Biol. Direct.

[17] Vahidi M., Aghakhani S., Martín D., Aminzadeh H., Kaveh M. (2023). Optimal band selection using evolutionary machine learning to improve the accuracy of hyper-spectral images classification: A novel migration-based particle swarm optimization. J. Classif..

[18] Mohammadrezaei E., Behravan M., Sarshartehrani F., Gračanin D. VividVR: Real-Time Optimization of Dynamic Light Probe Placement for Enhanced Visual Fidelity in VR Environments; Using SVM and Random Forest under Changing Conditions. Proceedings of the 2024 IEEE International Symposium on Mixed and Augmented Reality Adjunct (ISMAR-Adjunct).

[19] Awad M., Khanna R., Awad M., Khanna R. (2015). Support Vector Regression. Efficient Learning Machines: Theories, Concepts, and Applications for Engineers and System Designers.

[20] Sarkar D., Bali R., Sharma T. (2018). Practical Machine Learning with Python.

[21] Ho T.K. (1998). The random subspace method for constructing decision forests. IEEE Trans. Pattern Anal. Mach. Intell..

[22] Abdollahpour S.S., Huyen R.B., Le T.K., Nasri A., Hankey S. (2024). Built Environment’s Nonlinear Effects on Mode Shares Around BRT and Rail Stations. Transportation Research. Part D: Transport and Environment.

[23] Breiman L. (1996). Bagging predictors. Mach. Learn..

[24] Friedman J.H. (2001). Greedy function approximation: A gradient boosting machine. Ann. Stat..

[25] Specht D.F. (1991). A general regression neural network. IEEE Trans. Neural Netw..

[26] Willmott C.J., Matsuura K. (2005). Advantages of the mean absolute error (MAE) over the root mean square error (RMSE) in assessing average model performance. Clim. Res..

[27] Masrourisaadat N., Sedaghatkish N., Sarshartehrani F., Fox E.A. (2024). Analyzing quality, bias, and performance in text-to-image generative models. arXiv.

[28] Zhao D., Raja Reddy K., Kakani V., Read J., Carter G. (2003). Corn (*Zea mays* L.) growth, leaf pigment concentration, photosynthesis and leaf hyperspectral reflectance properties as affected by nitrogen supply. Plant Soil.

[29] Daughtry C.S., Walthall C., Kim M., De Colstoun E.B., McMurtrey Iii J. (2000). Estimating corn leaf chlorophyll concentration from leaf and canopy reflectance. Remote Sens. Environ..

[30] Peñuelas J., Filella I., Biel C., Serrano L., Save R. (1993). The reflectance at the 950–970 nm region as an indicator of plant water status. Int. J. Remote Sens..

[31] Katsoulas N., Elvanidi A., Ferentinos K.P., Kacira M., Bartzanas T., Kittas C. (2016). Crop reflectance monitoring as a tool for water stress detection in greenhouses: A review. Biosyst. Eng..

[32] Genc L., Demirel K., Camoglu G., Asik S., Smith S. (2011). Determination of plant water stress using spectral reflectance measurements in watermelon (*Citrullus vulgaris*). Am. Eurasian J. Agric. Environ. Sci..

[33] Peng Y., Fan M., Bai L., Sang W., Feng J., Zhao Z., Tao Z. (2019). Identification of the best hyperspectral indices in estimating plant species richness in sandy grasslands. Remote Sens..

[34] Raddi S., Giannetti F., Martini S., Farinella F., Chirici G., Tani A., Maltoni A., Mariotti B. (2022). Monitoring drought response and chlorophyll content in Quercus by consumer-grade, near-infrared (NIR) camera: A comparison with reflectance spectroscopy. New For..

[35] Tucker C.J. (1979). Red and photographic infrared linear combinations for monitoring vegetation. Remote Sens. Environ..

[36] Jackson R.D., Idso S., Reginato R., Pinter P. (1981). Canopy temperature as a crop water stress indicator. Water Resour. Res..

[37] Gitelson A.A., Gritz Y., Merzlyak M.N. (2003). Relationships between leaf chlorophyll content and spectral reflectance and algorithms for non-destructive chlorophyll assessment in higher plant leaves. J. Plant Physiol..

[38] Baker N.R. (2008). Chlorophyll fluorescence: A probe of photosynthesis in vivo. Annu. Rev. Plant Biol..

[39] Prasanna B.M., Cairns J.E., Zaidi P., Beyene Y., Makumbi D., Gowda M., Magorokosho C., Zaman-Allah M., Olsen M., Das A. (2021). Beat the stress: Breeding for climate resilience in maize for the tropical rainfed environments. Theor. Appl. Genet..

[40] Tardieu F., Draye X., Javaux M. (2017). Root water uptake and ideotypes of the root system: Whole-plant controls matter. Vadose Zone J..

[41] Yoder B.H. (2018). Corn yield following a delayed application of nitrogen. Ph.D. Thesis.

[42] Barzin R. (2021). Multispectral in-Field Sensors Observations to Estimate Corn Leaf Nitrogen Concentration and Grain Yield Using Machine Learning. Ph.D. Thesis.

[43] Clothier B.E., Green S.R. (1997). Roots: The big movers of water and chemical in soil. Soil Sci..

[44] Weaver J.E., Weaver J.E., Jean F.C., Crist J.W. (1922). Development and Activities of Roots of Crop Plants: A Study in Crop Ecology.

[45] Mueller S.M., Vyn T.J. (2016). Maize plant resilience to N stress and post-silking N capacity changes over time: A review. Front. Plant Sci..

[46] Li L., Sali A., Liew J.T., Saleh N.L., Ali A.M. (2024). Machine Learning for Peatland Ground Water Level (GWL) Prediction via IoT System. IEEE Access.

[47] Ding J., Yang A., Wang J., Sagan V., Yu D. (2018). Machine-learning-based quantitative estimation of soil organic carbon content by VIS/NIR spectroscopy. PeerJ.

[48] Mouazen A.M., Al-Asadi R.A. (2018). Influence of soil moisture content on assessment of bulk density with combined frequency domain reflectometry and visible and near infrared spectroscopy under semi field conditions. Soil Tillage Res..

[49] Cheng M., Jiao X., Liu Y., Shao M., Yu X., Bai Y., Wang Z., Wang S., Tuohuti N., Liu S. (2022). Estimation of soil moisture content under high maize canopy coverage from UAV multimodal data and machine learning. Agric. Water Manag..

[50] Lindner C., Bromiley P.A., Ionita M.C., Cootes T.F. (2014). Robust and accurate shape model matching using random forest regression-voting. IEEE Trans. Pattern Anal. Mach. Intell..

[51] Guan Y., Grote K. (2023). Assessing the Potential of UAV-Based Multispectral and Thermal Data to Estimate Soil Water Content Using Geophysical Methods. Remote Sens..

[52] Zhu S., Cui N., Guo L., Jin H., Jin X., Jiang S., Wu Z., Lv M., Chen F., Liu Q. (2024). Enhancing precision of root-zone soil moisture content prediction in a kiwifruit orchard using UAV multi-spectral image features and ensemble learning. Comput. Electron. Agric..

[53] Thevs N., Peng H., Rozi A., Zerbe S., Abdusalih N. (2015). Water allocation and water consumption of irrigated agriculture and natural vegetation in the Aksu-Tarim river basin, Xinjiang, China. J. Arid Environ..

[54] Zhu S., Cui N., Zhou J., Xue J., Wang Z., Wu Z., Wang M., Deng Q. (2023). Digital mapping of root-zone soil moisture using UAV-based multispectral data in a kiwifruit orchard of northwest China. Remote Sens..

